# Damage evolution of bi-body model composed of weakly cemented soft rock and coal considering different interface effect

**DOI:** 10.1186/s40064-016-1942-x

**Published:** 2016-03-08

**Authors:** Zenghui Zhao, Xianzhou Lv, Weiming Wang, Yunliang Tan

**Affiliations:** State Key Laboratory of Mining Disaster Prevention and Control Co-founded by Shandong Province and the Ministry of Science and Technology, Shandong University of Science and Technology , Qingdao, 266590 China; College of Mining and Safety Engineering, Shandong University of Science and Technology, Qingdao, 266590 China; College of Civil Engineering and Architecture, Shandong University of Science and Technology, Qingdao, 266590 China

**Keywords:** Weakly cemented soft rock, Bi-material model, Bi-body model, Damage evolution, Interface effect

## Abstract

Considering the structure effect of tunnel stability in western mining of China, three typical kinds of numerical model were respectively built as follows based on the strain softening constitutive model and linear elastic–perfectly plastic model for soft rock and interface: R–M, R–C^s^–M and R–C^w^–M. Calculation results revealed that the stress–strain relation and failure characteristics of the three models vary between each other. The combination model without interface or with a strong interface presented continuous failure, while weak interface exhibited ‘cut off’ effect. Thus, conceptual models of bi-material model and bi-body model were established. Then numerical experiments of tri-axial compression were carried out for the two models. The relationships between stress evolution, failure zone and deformation rate fluctuations as well as the displacement of interface were detailed analyzed. Results show that two breakaway points of deformation rate actually demonstrate the starting and penetration of the main rupture, respectively. It is distinguishable due to the large fluctuation. The bi-material model shows general continuous failure while bi-body model shows ‘V’ type shear zone in weak body and failure in strong body near the interface due to the interface effect. With the increasing of confining pressure, the ‘cut off’ effect of weak interface is not obvious. These conclusions lay the theoretical foundation for further development of constitutive model for soft rock–coal combination body.

## Background

Western mines in China are mainly located in Jurassic and Cretaceous weakly cemented soft rock strata. Due to the special diagenetic environment, the soft rocks present special characteristics of weak cementation, easy weathering and easy disturbance which cause the extremely unstable mechanical behavior. Therefore, roadways are usually excavated in relatively stable coal seam resulting in the special structure feature of weakly cemented soft rock–coal in surrounding rock. The roof, coal seam and floor constitute a composite bearing system. Dynamic disasters such as roof shock and weak impact induced by tunnel excavation and mining disturbance are actually a result of the unstable deformation of the whole mechanical system caused by the interaction of geological bodies with different mechanical properties (Zhao et al. 2008). Thus, the structural effect and damage characteristics of surrounding rock–coal system are urgently to be solved for predicting dynamic disasters in soft rock mine.

Stability of the composite structure of rock–coal body is closely related to the comprehensive interaction between geologic bodies and the contact surface. At present, studies on the interaction between different geologic bodies at home and abroad are mainly obtained in fracture mechanics which focus on the crack propagation in the interface (Kishen and Singh 2001; Chen et al. 2006). In the discussion of bond strength at contact surface, Xie et al. (2008) and Yi et al. (2008, 2009, 2012) produced two types of composite structure of mortar–concrete, mortar–rock respectively, and detailed analyzed their differences of mechanical behavior in the pressure–shear condition. In addition, some constitutive models of contact interface for different bi-body mediums were put forward based on a number of experimental results. For example, the non-linear elastic model proposed by Clough and Duncan (1971), the nonlinear elastic–perfectly plastic model established by Brandt (1985), the softening constitutive model for the interface of polymer cement mortar–concrete body presented by Zhang et al. (2012), the nonlinear elastic–perfectly plastic model for soil–structure interface established by Ruan and Wu (2004), the rigid-plastic model for soil–concrete created by Yin et al. (1995), the elastic–viscoplastic model proposed by Qian et al. (2008), and the damage model for soil–structure interface presented by Hu and Pu (2003). Besides, Esterhuizen et al. (2001) and Kim (2007) established different strain softening models for interface, respectively. Although many available models for contact interface have been proposed at present, these models are established based on different experiments, which are difficult to be embedded in common software. In numerical simulation, a contact element without thickness was put forward by Goodman et al. (1968) in order to simulate the contact behavior between different geologic bodies. However, it can’t accurately reflect the nonlinear mechanical behavior of sticking, sliding and tearing due to the linear constitutive relation. A nonlinear contact element with thickness which overcomes the shortcomings of Goodman element was further proposed by Desai et al. (1985), but it is difficult to determine the thickness and mechanical parameters of contact element. In previous numerical studies (Zhao et al. 2014a, b) on the failure behavior of bi-body model, coal seam and rock are usually regard as one common bearing body which neglect the interface effect.

At present, the failure characteristics of coal and rock mass are mainly studied by indoor test. However, research on the damage evolution of coal–rock composite body is seldom reported. The only achievements are obtained based on a simple combination of coal and rock with a strong adhesive bond which is inconsistent with the actual situation. Actually, it is deficient in the sampling success rate and high cost of field testing for the study on weakly cemented soft rock–coal body. Based on the above shortcomings, in this paper, the numerical method is employed to discuss the failure evolution characteristics of weak cementation soft rock–coal formation. Two kinds of modes, namely, bi-material model and bi-body model were proposed considering different bonding states at contact interface. Their failure process were detailed analyzed in order to find out the unstable failure information which provide theoretical guidance for the construction of Western mine in China.

## Constitutive model of weakly cemented soft rock–coal composite structure

### Strain softening model for soft rock

The unstable failure of weakly cemented soft rock is a progressive process from microcracks extending to crack coalescence. The formation of failure zone under tri-axial compression is started at pre-peak stage, and shaped at post-peak stage or even at residual stage. The strain softening feature is a necessary condition for its shear failure.

**Fig. 1:**
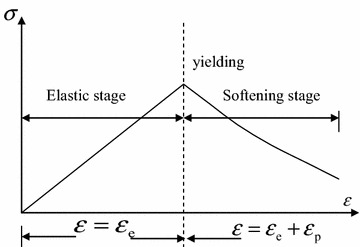
Stress–strain relation of weakly cemented soft rock

The strain softening constitutive model shown in Fig. 1 was employed. At pre-peak stage, only elastic strain *ε*_e_ produces in the element. After yielding, its total strain contains elastic strain and plastic strain, namely, *ε* = *ε*_e_ + *ε*_p_ where *ε*_p_ stands for the plastic strain.

The associated Mohr–Coulomb shear failure criterion and tensile failure criterion for the element yielding was adopted.

The M–C failure criterion can be described as

1 $$ F^{S} = \sigma_{1} - \sigma_{3} \tan^{2} (45^{ \circ } + \phi /2) + 2c\tan (45^{ \circ } + \phi /2) $$

and the tensile failure criterion is

2 $$ F^{t} = \sigma^{t} - \sigma_{3} $$

where *φ*, *c*, *σ*^t^ are the friction angle, cohesive and tensile strength of geologic body, respectively.

After yielding, the cohesive strength and friction angle of rock mass will be deteriorated. Here, equivalent plastic strain is chosen as the softening parameter, then

3 $$ c = c(\varepsilon^{\text{ps}} ),\quad \phi = \phi (\varepsilon^{\text{ps}} ), $$

and

4 $$ \begin{aligned} \varepsilon^{\text{ps}} & = \frac{1}{\sqrt 2 }\sqrt {\left( {\varepsilon_{ 1}^{\text{p}} - \varepsilon_{\text{m}}^{\text{p}} } \right)^{2} + \left( {\varepsilon_{\text{m}}^{\text{p}} } \right)^{2} + \left( {\varepsilon_{ 3}^{\text{p}} - \varepsilon_{\text{m}}^{\text{p}} } \right)^{2} } \\ \varepsilon_{\text{m}}^{\text{p}} & = \left( {\varepsilon_{ 1}^{\text{p}} + \varepsilon_{ 3}^{\text{p}} } \right)/3 \\ \end{aligned} $$

where $\varepsilon_{ 1}^{\text{p}}$ and $\varepsilon_{ 3}^{\text{p}}$ are both plastic principal strain component.

**Fig. 2:**
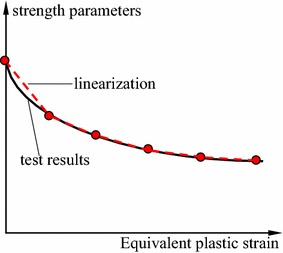
Strength deterioration at post-peak stage

From Eq. 3, the shear strength parameters can be determined by the iterative computation of plastic strain which have been detailed discussed based experimental results and theoretical model (Zhao et al. 2014a, b). If the shear strength parameters at post-peak stage has a non-linear relation with plastic strain as shown in Fig. 2, this pattern can be defined the method of subsection linearization. In each subsection, the change rule is approximately linear.

### Contact model of soft rock–coal composite structure

**Fig. 3:**
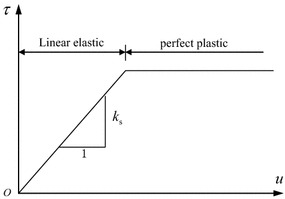
Elastic–plastic constitutive model of interface

Linear elastic–perfectly plastic model based on Mohr–Coulomb shear failure criterion was employed to simulate the contact behavior between soft rock and coal as shown in Fig. 3.

On the elastic stage, the normal force *F*_n_ and shear force *F*_si_ can be determined by the iterative computation of contact displacement *u*. Three contact states exist in different deformation stages: sticking state, sliding state and separation state. If contact state is in plastic flow state, the following relation should be satisfied

5 $$ \begin{aligned} F_{\text{smax}} & = cA +  \tan \phi \left( {F_{\text{n}} - pA} \right) \\ F_{\text{n}} & = \sigma_{\text{t}} \\ \end{aligned} $$

where *p* represents the pore pressure, and *A* stands for contact area.

1. If *F*_n_ < σ_t_ and *F*_si_ < *F*_smax_, the interface is in sticking state, and the nodal displacement is at linear stage.
2. If *F*_n_ < σ_t_ and *F*_si_ = *F*_smax_, the interface is in plastic flow state.
3. If *F*_n_ > σ_t_, the geologic bodies on two sides of interface are in separation state.

## Conception of bi-material model and bi-body model

### Calculation model

**Fig. 4:**
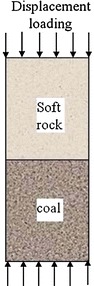
Compression model of coal–rock combined body

According to the indoor test, let the diameter and height of soft rock–coal body be 50 mm and 100 mm, respectively, and the height ratio is 1 as shown in Fig. 4. Only axial motion was allowed at the upper and lower end face, other directions were constrained. The model was loaded by displacement at a speed of 2 × 10^−5^mm/step. On the basis of test results, mechanical parameters of soft rock and coal were set in Table 1.

**Table 1:** Physico-mechanics parameters of specimens

Medium	Elastic modulus (GPa)	Poisson’s ratio	Cohesion (MPa)	Friction angle (°)	Tensile strength (MPa)
Soft rock	2.1	0.252	3.5	44	0.3
Coal	1.5	0.272	2.5	40	0.5

**Fig. 5:**
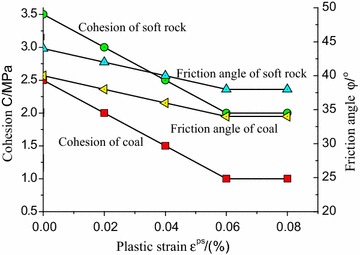
Attenuation laws of strength parameters of coal and rock

Considering the strain softening behavior of soft rock and coal, the linearization was processed in the attenuation rule of strength parameters according to test results (see Fig. 5).

Let R, M and C represent soft rock, coal and contact interface, respectively. For different bonding state, the following models can be established: (1) the composite structure dose not contain contact interface denoted as R–M model; (2) the composite structure contains contact interface with high bonding strength denoted as R–C^s^–M; (3) the composite structure contains contact interface with low bonding strength denoted as R–C^w^–M.

**Fig. 6:**
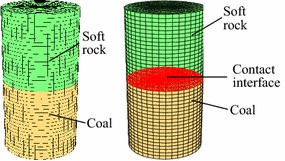
R–M model and R–C–M model

Damage behaviors of the three models were compared based on different modeling approaches and interface parameters. The contact parameters for strong bonding and weak bonding were set as shown in Table 2. The stiffness parameters can be calculated as

6 $$ k_{\text{n}} = k_{\text{s}} = 10\,{ \hbox{max} }\left\{ {\frac{{E\left( {1 - \nu } \right)}}{{\left( {1 - 2\nu } \right)\left( {1 + \nu } \right)}} \cdot \frac{1}{{\Delta n_{ \hbox{min} } }}} \right\} $$

where *E* and ν are respectively the elastic modulus and Poisson’s ratio of the hardest geologic body, Δ*n*_min_ is the minimum element size of the contact area in normal direction. Obviously, the smaller the minimum element size, the higher the calculation stiffness of contact interface. The two different models are shown in Fig. 6.

**Table 2:** Mechanical parameters of contact interface under different bonding states

Contact state	Normal stiffness (GPa m^−1^)	Tangential stiffness (GPa m^−1^)	Cohesion (MPa)	Friction angle (°)
Strong	100	100	100	40
Weak	10	10	1	40

### Failure behavior of different models

**Fig. 7:**
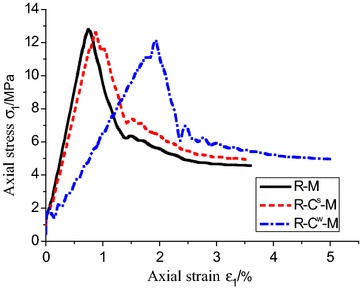
Stress–strain curves of different mechanical models

Figure 7 illustrates the stress–strain relations of soft rock–coal composite structure under different mechanical models. For R–C^s^–M model and R–M model, the evolution law of stress and strain is very similar, and the peak stress is at the fundamental superposition. The stiffness of R–C^s^–M at pre-peak stage is slightly lower than the R–M model, while the stress drop at the post-peak stage is slightly higher. However, the peak strength of R–C^w^–M model was lower than that of above two models, and the shift is apparent, resulting in a greater strain. This shows that the strain softening behavior of the model is more serious.

**Fig. 8:**
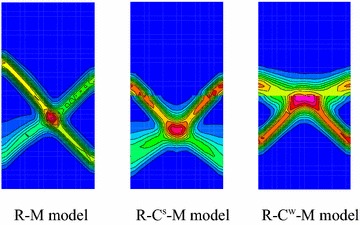
Shear bands of different mechanical models

Figure 8 shows the shear failure modes of the combined model under different models. For R–M model, it presents overall plastic shear failure and two asymmetric conjugate shear bands, this result from the integral grid system which neglects the contact surface. Two symmetrical shear bands start at the middle of the coal body across the interface and extend to the rock body in R–C^s^–M model. Due to the strong bonding, the model also exhibits continuous failure, but the length of the shear band is short, and the damage range of the rock is smaller compared to R–M model. In R–C^w^–M model, two symmetrical shear zones starting from contact interface extend to the middle of coal body. However, they are unable to across the interface and extend to the rock due to the weak bonding strength. From the above results, it presents continuous failure behavior in R–M model and R–C^s^–M model which can be defined as bi-material model, while damage is concentrated in weak media in R–C^w^–M model which is regarded as bi-body model.

## Damage evolution law of bi-body model composed of weakly cemented soft rock and coal

### Calculation condition

The three-dimensional cylinder with a diameter of 50 mm and height of 100 mm was established. The model was meshed uniformly into 20 elements in the radial direction and 40 elements in the axial direction, respectively. A confining pressure of 2 MPa was applied on the lateral circumference. Only axial motion was allowed on the loading face and other directions were constrained. The physico-mechanics parameters and the post-peak attenuation of soft rock and coal is set the same with section 3.1. The interface parameters were set as shown in Table 3. Loading sequences for the model are: firstly, the confined pressure of 2 MPa was applied, followed by deformation velocity on the upper and lower end face with opposite direction to simulate the triaxial compression test.

**Table 3:** Mechanical parameters of contact interface under different stick states

Model	Normal stiffness (GPa m^−1^)	Tangential stiffness (GPa m^−1^)	Cohesion (MPa)	Friction angle (°)
R–C^s^–M	50	50	2	40
R–C^w^–M	10	10	1	40

**Fig. 9:**
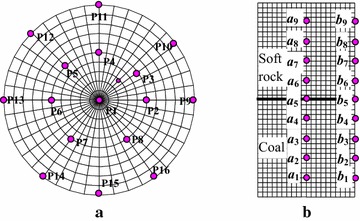
Arrangement of watching points. **a** Watching point on the interface, **b** watching point in the axial direction

The model was loaded by displacement at a speed of 1 × 10^−7^ m/s. The servo control method is used in order to avoid the inertia effect caused by loading rate. The loading velocity of both models is continuously adjusted according to the value range of the maximal unbalanced force. This process would lead to stress fluctuation around its initial stage and the peak point. In order to monitor the failure characteristics, a group of watching points were set as follows: 16 points were evenly arranged at the interface to monitor the axial displacement (as shown in Fig. 9a), and 9 points were set with equal interval in axial direction and edge along the axis to monitor the axial deformation velocity and displacement, respectively (see Fig. 9b). The value of shear strain rate, stress and strain during failure process can be extracted by the programming in Fish.

### Complete stress strain curve and deformation rate evolution of monitoring points

**Fig. 10:**
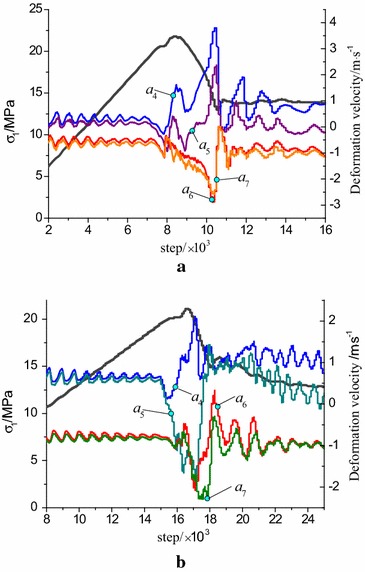
Changing of stress evolution with deformation velocity. **a** Bi-material model (R–C^s^–M), **b** bi-body model (R–C^w^–M)

The evolution relationship between stress and deformation velocity of monitoring points were shown in Fig. 10. Because the fluctuation range of the deformation velocity were gradually reduced with the increasing of the distance to the interface, the results of four typical monitoring points (a4, a5, a6, a7) around the interface were given.

Due to the inhomogeneous deformation caused by strain localization in numerical calculation, the two models both present stress fluctuations during the final elastic stage at the pre-peak stage. The bearing capacity was decreasing with the increase of deformation at post-peak stage which shows obvious strain softening behavior. Compared to the bi-material model, the peak stress of bi-body model which produced at a greater strain was apparently lower. Therefore, the bi-body model is more readily to appear even intense deformation under identical loading conditions.

The deformation velocities of the monitoring points present obvious fluctuation from pre-peak fluctuating stage to the post-peak residual stage, indicating that the models close to the interface zones are damaged violently. The abnormal jumps of deformation velocity exactly illustrate the approach of the main rupture. The velocity jump of monitoring points (*a*_4_ and *a*_5_) in R–C^s^–M model appeared three times from the initial stress fluctuation to the residual stage, where the first and second jump are negative corresponding to the pre-peak stage and post-peak strain-softening stage, respectively. These sudden jumps are due to the failure of the coal at the two loading points, where rock mass is in the elastic stage, and release elastic strain energy to the coal body. The third fluctuation jump is positive and greater than the former two, and the corresponding loading step is at the end of the strain softening stage. This appears in the later stage of strain softening where the main rupture in coal body is extended to the rock, and deformation velocities of rock mass also shows negative fluctuation due to the main rupture of the integral model.

The deformation velocity of monitoring points (*a*4 and *a*5) in R–C^w^–M model were basically the same at the initial elastic loading stage while deviated in the late stage. The first fluctuation of monitoring point a4 appears before the stress fluctuation point at pre-peak stage and the negative fluctuation is not obvious, while a5 presents obvious negative jump. At the strain softening stage, a large positive jump appears in point *a*4, while the monitoring points *a*5, a6 and a7 produce a large negative fluctuation. This indicates that the zones near the contact surface are suffered severe damage. From the above results, the failure evolution processes in the two models are completely different although the stress evolutions show the same changing law. The jumps of deformation velocities can be regarded as the precursor information of failure and a sign of the main rupture perforation.

### Evolution process of shear bands in combined model

#### Failure evolution process of R–C^s^–M model

**Fig. 11:**
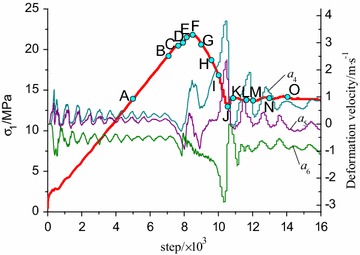
Step distribution of load monitoring for R–C^s^–M model

In order to reveal the whole failure process, 15 monitoring points were set in the stress curve according to Fig. 10a, as shown in Fig. 11. Among them, point A and B lie in the elastic stage, and point C is set at the starting of the stress fluctuation stage. The contour results of shear strain increment in the coal–rock combined model can be extracted by Fish program.

**Fig. 12:**
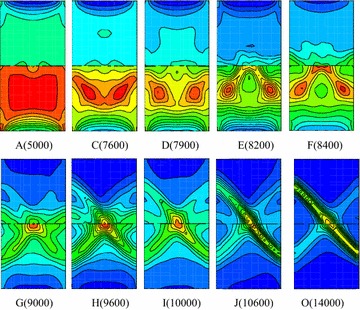
Contour of shear bands in coal–rock body (R–C^s^–M)

Figure 12 presents contours of plastic shear zone in axial symmetry plane of coal–rock body under different typical loading steps. When loading to point A, the distributions of shear strain in the two bodies are relatively homogeneous. However, a degree of soft deformation appears at the upper and lower end face due to the restraining effect at the end face. When loading to point C, the shear strains become inhomogeneous. It is obvious higher in the middle of coal body appearing as V-shaped in shear strain concentration area, and the end face effect intends to weaken. This step is just at the starting of the stress fluctuation in elastic stage and also the loading point when deformation velocity presents fluctuation. When the loading step is reached to point E, the shear strain zone in coal presents an inverted V shape with the middle of the interface as its vertexes.

When loading to point F, two obvious shear bands appeared in coal body, and the shear strain rate in bands is higher than that of outside. Due to the restraining effect, partial softening deformation appears in rock body near the interface, and the deformation velocity presents positive jump according to Fig. 10a. When continuing to load into point G at post-peak stage, the shear bands are extended into rock body and presents “butterfly” shape around the middle part of the interface. At this step, the softening zone at the upper end face disappears. The deformation velocity is yielded negative fluctuation due to the elastic-rebound of rock body which caused by failure of coal and rock near the interface. When loading to point H, the two shear bands are developed unsymmetrical: one shows the tendency of extending to rock body, while the other remains unchanged, and this tendency is more obvious when loading into point I. The main shear bands are developed to the middle of left rock when loading to point J. However, the shear strain rate within the bands still mainly concentrated nearby the coal body and interface, while the other shear band tends to become indistinct. This loading step is just at the lowest of the post-peak stage where deformation velocities of coal and rock present obviously positive and negative fluctuations, respectively. From point K to point O, the shear strain is developed mainly within the shear bands, the other one become indistinct, and the model is entered into residual deformation stage. Finally, a spatial shear band is formed from the middle of right coal body to the middle of left rock, namely, strain localization zone. The model exhibits plastic shear along the shear band under three-dimensional compression. It is clear that each evolution stage of the shear bands are exactly corresponds to the deformation velocity fluctuation and stress fluctuation as shown in Fig. 10.

#### Failure evolution process of R–C^w^–M model

**Fig. 13:**
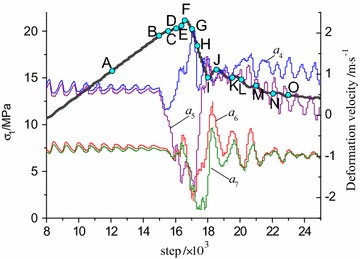
Step distribution for load monitoring for R–C^w^–M model

As is shown in Fig. 13, 15 monitoring points were also arranged in the stress curve according to Fig. 10b in order to analyze the failure process of R–C^w^–M model.

**Fig. 14:**
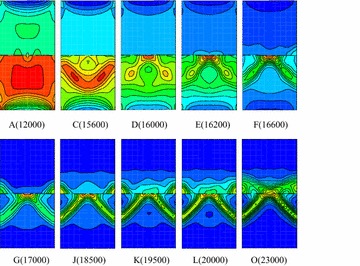
Contour of shear bands of coal-rock body (R–C^w^–M)

When loading to point A, the shear strain increment in coal body is significantly greater than that of rock, and the upper and end face of coal body shows strain softening phenomenon. When loading to point C, namely the starting location of stress fluctuation at pre-peak stage, a V-shape shear band appears in the middle of coal body. The deformation velocity is yielded negative fluctuation, while shear strain bands in rock body remains uniformly, and restriction on end face is weakened (Fig. 14).

At loading point D, the shear bands pattern in coal body is evolved from the original V-shaped into an inverted V shape with the middle of the interface as its vertexes. At loading point E, a V-shaped shear band has been preliminarily formed. Compared with the velocity variation, the interface cohesion is weaker during the pre-peak stress fluctuation stage, so the shear strain in coal body is changed more intensely in the middle of the interface and the deformation velocity of monitoring points present greatly negative fluctuation. When loading to point F, the inverted V-shaped shear bands evolved from the middle of the interface exhibit evidently and the restrictions on end face disappear. When loading to point G at post-peak stage, two local shear zones appear in the both sides of the interface and the shear bands become thin due to the interface effect. At loading point H, I and J, the shear bands pattern remain unchanged and just the shear strain increment within the bands is further increased. When loading to point K, shear strain increment in rock body near the interface begin to increase. Thereafter, the development of shear bands is mainly concentrated on the rock nearby the interface from the results at load point L, M and N. When loading to point O, a penetrating band is formed.

From the failure process, it presents general shear failure along a penetrating band from coal to rock in R–C^s^–M model due to the higher interface strength. Because of the weak interface effect, the failure in R–C^w^–M model is started from the middle interface, and then developed into two V-shaped shear bands (with two slide surfaces in spatial) in coal body.

However, these shear bands are unable to pass through the interface. Besides, stress state of the rock near the interface is changed and the strength is weakened. Thus, it presents local shear failure in X-shaped of coal body and general transverse shear failure along the zone nearby the interface in R–C^w^–M model.

#### Displacement evolution of watching points

The displacement variations in the loading direction at the contact interface can well reflect the failure characteristic of coal–rock body which includes the failure information. Displacement evolutions of the interface can be obtained through 3D surface interpolation in MATLAB according to the axis displacement results of watching points showed in the Fig. 10a.

**Fig. 15:**
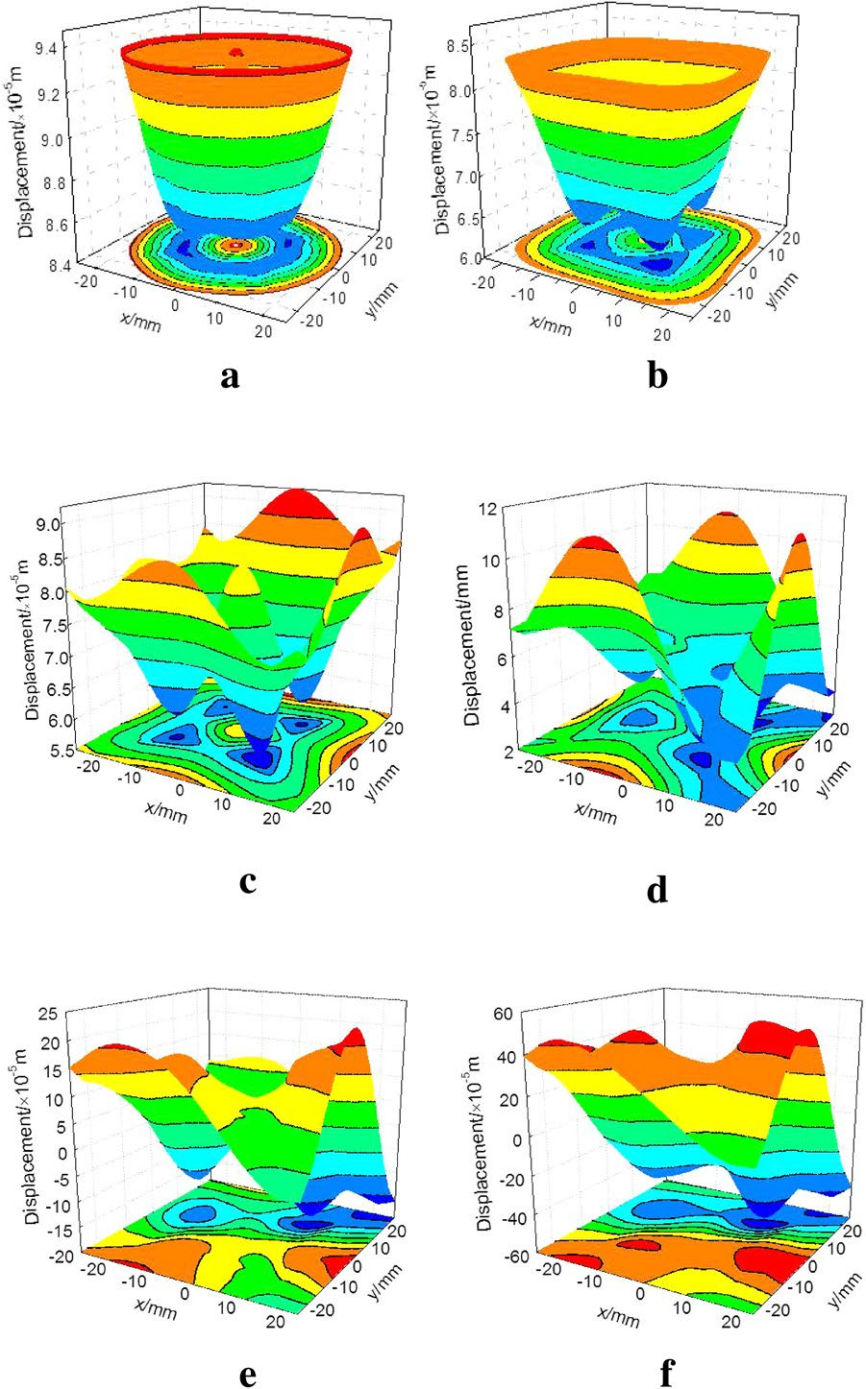
Axial displacement of contact interface (R–C^s^–M). **a** Step 7900, **b** step 8200, **c** step 8400, **d** step 9000, **e** step 10000, **f** step 12000

Figure 15 shows the contour results of displacement evolutions in R–C^s^–M model. At the elastic stage, that is, before the step 7900, the displacement is distributed uniformly from the middle to the edge of the interface. When loading to the step 8200, the displacements from the middle to the edge are gradually reduced caused by the local shear band in the middle of the interface. However, the interface deformation remains symmetric. At the peak point (step 8400), the displacements become highly asymmetric due to the inhomogeneous failure. At load step 9000, the model is yielded asymmetric deformation and the axial deformation of certain elements appears negative fluctuation. Thereafter, the axial deformation of the interface presents asymmetric distribution. Owing to the further development and perforation of the failure bands, the differences of axial displacements are gradually increased. For instance, the maximum displacement differences at step 100000 and 120000 are 0.04 and 0.1 mm, respectively. Thus, the asymmetric distribution of the axial displacement and the negative fluctuation of the watching points also contain the failure information.

**Fig. 16:**
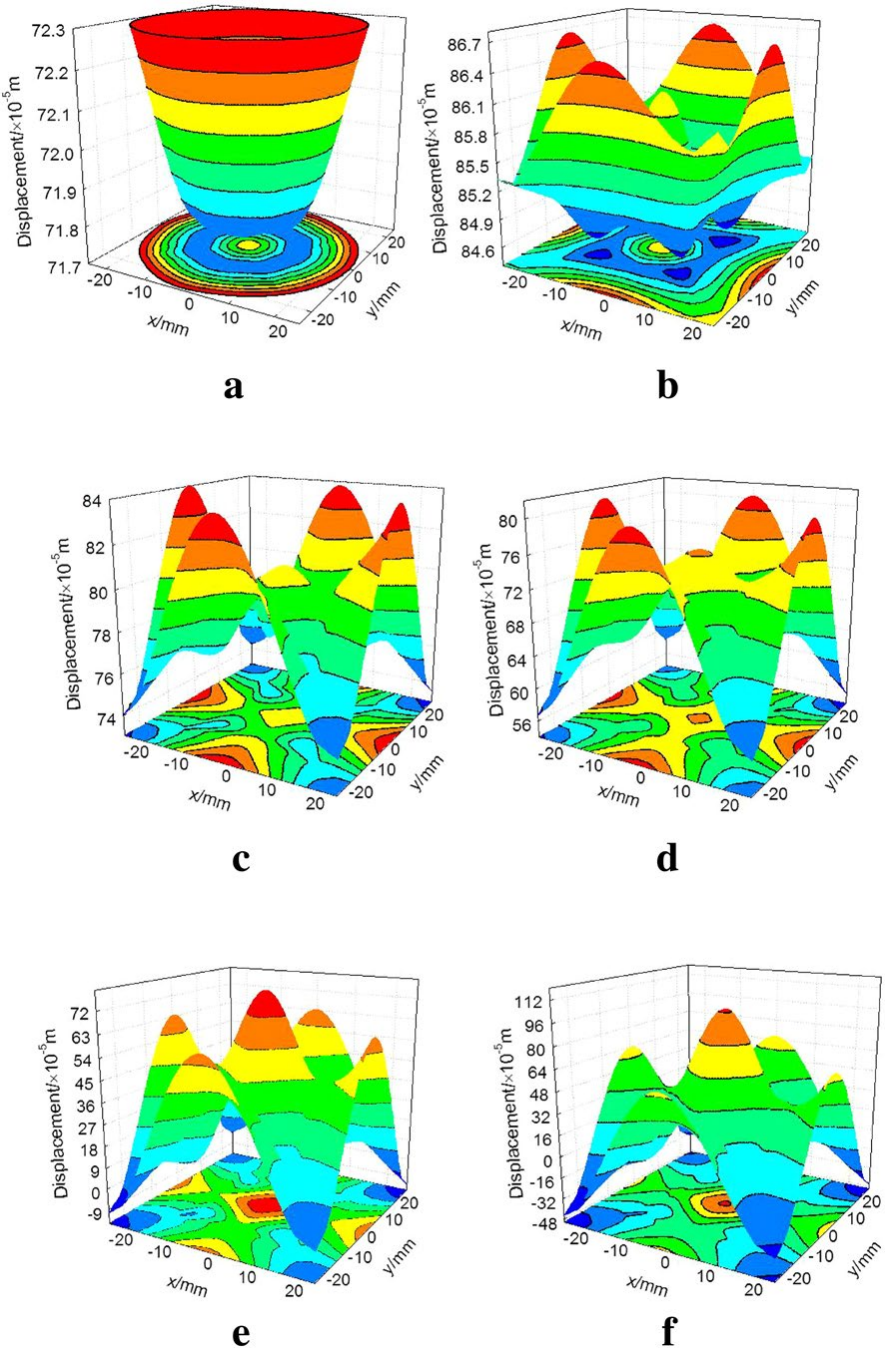
Axial displacement evolution of contact interface between coal and rock (R–C^w^–M). **a** Step 15000, **b** step 16000, **c** step 16200, **d** step 16600, **e** step 17400, **f** step 19500

Figure 16 presents the contour of axial displacement in R–C^w^–M model. When the model is loaded on the elastic stage, the axial displacements are distributed basically uniformly, and the maximum displacement difference is only 0.006 mm. The homogeneous degree of the displacement is reduced at step 16000. When loading to step 16200, the displacements are gradually increased from edge to inner with symmetric distribution. At the peak point (step 16600), the displacement differences on the interface are increased. The displacement of each monitoring point presents obviously asymmetric distribution owing to the shear bands.

**Fig. 17:**
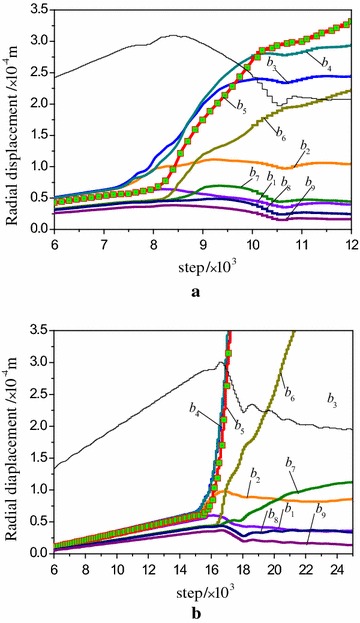
Radial displacement evolution of watching points. **a** R–C^s^–M model, **b** R–C^w^–M model

Figure 17 demonstrates the radial displacement variation of the watching points. As the deformation in coal body is greater than that in rock at the elastic stage, the displacements are both along the forward coordinate axis in two models. Before the first stress-drop at pre-peak stage, the radial displacements are slowly increased at a stable speed, and the displacement of each watching point is not separate. At stress-drop stage, the displacements of the interface, coal and rock near the interface present positive fluctuation especially severely in the interface and the coal body.

From the above analysis, this location is just the loading point where presents obvious shear band in coal body, so it can be used as the precursory information of the model rupture. The fluctuation of displacement in R–C^w^–M model is higher than that of R–C^s^–M model. Moreover, the axial deformation of the watching points b1, b2, b7, b8 and b9 which close to the end face remain small during the loading process due to the restriction effect at the end face.

#### Failure behavior of the combined model under different stress states

**Fig. 18:**
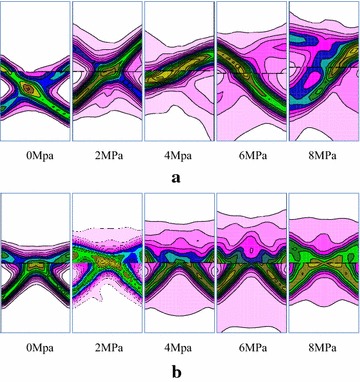
Plastic failure under different confining pressures. **a** R–C^s^–M model, **b** R–C^w^–M model

Figure 18a demonstrates the comparison of shear bands under different confining pressures for R–C^s^–M model. There are two asymmetry shear zones under uni-axial compression, one is the main shear band, and the other is not obvious. The Shear zone is developed from the right-bottom side of the coal body and extended to the left-bottom of the rock mass. With the confining pressure increasing to 2 MPa, the distribution of shear bands are still differentiated clearly and the overall failure behavior is enhanced. Only one shear zone appears in the model when the confining pressure is increased to 4 MPa, and its width is further enlarged. The decreasing of penetrating area indicates that the strength of the combined model is improved. When the confining pressure is increased to 6 MPa, the shear bands present good continuous pattern, and the model shows overall shear failure and typical unstable characteristics of bi-material model. The shear bands become blur when the confining pressure is raised to 8 MPa, and a large area of plastic zone appeared in the combined model. The model presents local shear and whole plastic failure. From the failure evolution, the strength of combined model was enhanced due to the increasing of confining pressure, especially for the coal body. If the confining pressure is lower than 4 MPa, the model also presents overall failure characteristics, but the shear strain is not continuous in the vicinity of the interface. When the confining pressure exceeds 4 MPa, the “cut off” effect of the interface is weakened due to the high pressure, and failure is mainly depending on the strength of geologic bodies.

Figure 18b demonstrates the damage behavior of R–C^w^–M model under different confining pressures. The failure pattern of combined model presents the same inverted “V” type with the increasing of confining pressures, but its width is increased. Besides, the shear plastic zone in the rock near the interface is gradually increasing. The shear zone is gradually extended to the middle part of interface under the lower confining pressure and connected together when the confining pressure is increased to 8 MPa. The combined model presents shear failure of coal body and the plastic failure of rock near the interface. The “cut off” effect of interface is not obviously weakened under high confining pressure which is different from the R–C^s^–M model. The sub layer in the composite model does not present the overall failure characteristic.

## Conclusion

According to the composite fabric feature of weakly cemented soft rock–coal, a calculating model for two-element combined model is established. Then the failure behavior and stress evolution considering different interface effects were detailed analyzed based on numerical simulation, the main findings are as follows:

1. For the combined model without consideration of interface effect or with strong bonding, it presents continuous failure along an shear band which is started form the weak geologic body and extended to the strong body. This model can be defined as bi-material model. The “cut off” effect of interface is weakened with the increasing of confining pressure.
2. For the combined model with weak bonding, it presents partial failure in weak body along shear band with inverted “V” type, and this model can be defined as bi-body model. Displacements of the monitoring points on the interface appear obvious uneven distribution due to the localized deformation. The “cut off” effect of interface is not obvious under high confining pressure.
3. The failure evolution of the composite model shows a good correspondence with the deformation rate of the rock mass near the interface, and failure information can be captured accordingly. The first jump point of the deformation rate demonstrates the initiation of the main rupture, and can be regarded as the precursor information of the failure. The second jump point represents the perforation of the main rupture. The amplitude of the sudden jump is violent in whole failure wave process, so it can be recognized.
4. The failure characteristic of surrounding rock in underground engineering and roof and floor in mines should be analyzed based on the bearing capacity system of the combined surrounding rock and roof-coal-floor. Among them, the failure of each medium is not only depends on the self-strength, but also on the contact interface bonding of other surrounding media and stress state.
